# Room-Temperature Pressure-Induced Optically-Actuated Fabry-Perot Nanomechanical Resonator with Multilayer Graphene Diaphragm in Air

**DOI:** 10.3390/nano7110366

**Published:** 2017-11-04

**Authors:** Cheng Li, Tian Lan, Xiyu Yu, Nan Bo, Jingyu Dong, Shangchun Fan

**Affiliations:** 1 School of Instrumentation Science and Opto-Electronics Engineering, Beihang University, Beijing 100191, China; lantian435@163.com (T.L.); yuxiyu@buaa.edu.cn (X.Y.); shangcfan@buaa.edu.cn (S.F.); 2 Science and Technology on Metrology and Calibration Laboratory, Beijing 100095, China; 3 Beijing Institute of Automatic Control Equipment, Beijing 100074, China; andynan_bo@sina.com (N.B.); djybao@163.com (J.D.)

**Keywords:** multilayer graphene diaphragm, Fabry-Perot nanomechanical resonator, air damping, pressure sensitivity, room temperature

## Abstract

We demonstrated a miniature and in situ ~13-layer graphene nanomechanical resonator by utilizing a simple optical fiber Fabry-Perot (F-P) interferometric excitation and detection scheme. The graphene film was transferred onto the endface of a ferrule with a 125-μm inner diameter. In contrast to the pre-tension induced in membrane that increased quality (*Q*) factor to ~18.5 from ~3.23 at room temperature and normal pressure, the limited effects of air damping on resonance behaviors at 10^−2^ and 10^5^ Pa were demonstrated by characterizing graphene F-P resonators with open and micro-air-gap cavities. Then in terms of optomechanical behaviors of the resonator with an air micro-cavity configuration using a polished ferrule substrate, measured resonance frequencies were increased to the range of 509–542 kHz from several kHz with a maximum *Q* factor of 16.6 despite the lower Knudsen number ranging from 0.0002 to 0.0006 in damping air over a relative pressure range of 0–199 kPa. However, there was the little dependence of *Q* on resonance frequency. Note that compared with the inferior F-P cavity length response to applied pressures due to interfacial air leakage, the developed F-P resonator exhibited a consistent fitted pressure sensitivity of 1.18 × 10^5^ kHz^3^/kPa with a good linearity error of 5.16% in the tested range. These measurements shed light on the pre-stress-dominated pressure-sensitive mechanisms behind air damping in in situ F-P resonant sensors using graphene or other 2D nanomaterials.

## 1. Introduction

Nanomechanical resonators have been widely used to measure force [1], mass [2], charge [3], and displacement [4] with high sensitivity. Currently, silicon is a primary material for fabricating precision micro- and nanoelectromechanical systems (NEMS), which enables the production of devices with both high sensitivity and large bandwidths [5]. However, recently two-dimensional (2D) nanomaterials, such as graphene and molybdenum disulfide (MoS_2_), have been enabling a new class of atomically thin resonators for sensing and actuation operations because of the vanishing of bending rigidity with decreasing thickness and excellent mechanical properties [6,7,8,9,10]. Among the 2D materials used for the development of these devices, graphene has attracted the most attention and has been extensively studied by far [10,11,12,13], in view of its high stiffness, strength, and thermal conductivity along the basal plane. Particularly, Bunch et al. transferred suspended single- and multi-layer graphene sheets over trenches and measured the fundamental frequency of 1–170 MHz and a *Q* factor of 20–850 at room temperature and a pressure of <10^−6^ Torr [10]. Since then, more efforts have been made to investigate the resonance behaviors of NEMS devices made of single-layer, few-layer, or multilayer graphene membranes at room temperature and vacuum pressure by employing various graphene configurations such as nanoribbon [11], square and circular membranes [12,13], as well as graphene fabrication schemes [14,15], along with free-space optical or electrical actuation and detection techniques [13,16,17].

These aforementioned experimental studies have significantly boosted the understanding of graphene resonance characteristics. However, in these experiments, graphene flakes are generally mechanically exfoliated by means of well-controlled forces [10,11,14], and their dimensions are limited to the order of several μm. It is important to point out that the motions of sensitive diaphragms in these resonators are commonly detected by electrical or optical readout under vacuum conditions. Although it is well known that electrical detection is vital for the integration of these devices, and is attractive for many applications [11], optical actuation represents an approach of direct coupling energy into suspended devices without the use of additional thin film layers [18]. Furthermore, the strong light-matter interaction in ultrathin 2D materials leads to extremely large absorption coefficients, enabling the aforementioned resonance phenomena to be investigated using relatively simple photothermal detection and excitation scheme [19]. Therefore, simple, miniature and in situ optical actuation of graphene film in a sample is vital for fabricating a resonant sensor, and then in situ grasping its resonance behaviors. Unfortunately, currently a complicated free-space optical actuation approach is commonly used in the previously reported literatures, where numerous specific measurement setups are typically necessary, such as a beam expander, a lens, a dichroic mirror, and etc. [20]. In addition, the sample test process is generally time-consuming due to the optical path adjustment. In other words, this type of approach mentioned above is more appropriate for laboratory tests instead of in situ measurement. Recently, Ma et al. constructed a compact ferrule-top nanomechanical resonator using beam-shaped multilayer graphene diaphragm with the thickness varying from 10 to 20 nm, which demonstrated the resonant frequencies of 60–204 kHz and *Q* values of 81–103 at room temperature in the vacuum of 1 × 10^−4^ mbar [21]. It should be noted that the resonant frequency corresponding to a fundamental resonant mode was observed as a function of surrounding gas pressure in a vacuum instead of pressure difference because of the existence of a conductive cavity. The similar conductive cavity configuration to suspend graphene film was also utilized in [22], wherein a frequency shift of 4 MHz between 8 and 1000 mbar with a *Q* factor less than 100 was observed for an exfoliated suspended few-layer graphene flake with a smaller diameter of 5 μm and a thickness of ~10.5 nm by an free-space interferometry setup. Due to zero pressure difference at both sides of the suspended membrane, the resonators with venting gas channels typically operate at lower pressures than a normal pressure, which also correspond to a comparatively low vibration damping condition. For instance, viscous damping is not the dominant dissipation process for the resonator reported in [22] because the Knudsen number, *K*_n_ = *λ*_MFP_/*l*_device_ [23], where *λ*_MFP_ is the mean free path of air molecules and *l*_device_ is the graphene device characteristic length, ranges from 0.01 to 10. However, in reality, air damping is a significant viscous dissipation mechanism when nanomechanical resonators are operated in a moderate vacuum or near ambient conditions. Moreover, resonance characteristics in air micro-cavity also completely differ from those in a vacuum chamber [24,25]. Although preliminary studies under vacuum (low damping) conditions conducted by Bunch et al. on electromechanical resonators from mechanically-exfoliating graphene sheets over trenches in SiO_2_ have found that the tension resulting from the fabrication process could increase the resonant frequency [10], graphene typically has higher energy loss [26], especially in air damping owing to a lower thickness per unit layer and mass density. This suggests that the overall effects of the tension in membrane and viscous air damping on resonance characteristics should be estimated for improving the clamping at the suspended graphene-substrate interface. For these reasons, the pre-stress-dependent pressured-induced bulging of graphene membrane in air damping may result in valuable resonance characteristics with varying pressure differences.

Hence, in this paper, from the viewpoint of in situ applications of graphene-based F-P resonant sensors, we developed a simple and miniature micro-air-gap-based optical fiber F-P resonator covered with a chemical vapor-deposited (CVD) ~13-layer graphene circular diaphragm instead of beam-shaped diaphragm in [21] in order to study the pressure-induced membrane vibration behaviors in air at room temperature. The use of circular diaphragm is due to the fact that clamping graphene membranes on all sides contributes to suppressing the variation in resonance frequency and then making their motion behaviors more predictable [12]. Additionally, the intricate and time-consuming post-processing using a femtosecond laser in [21] can be avoided. It is worth mentioning that the film was suspended onto a standard ZrO_2_ ferrule endface with a diameter of 125 μm that is by far larger than several microns in previously reported resonators [10,11,12,13,14,15,16,17,19,22], which helps the packaging design and in situ performance optimization of this type of F-P resonator. Additionally, an open F-P cavity made of a conventional ZrO_2_ mating sleeve was herein fabricated to make the comparative analysis of pre-stress-dependent resonance behaviors. Then, the resonance performance with varying pressure differences ranging from 0 to 199 kPa was tested by the presented simple optical fiber F-P interferometric resonance readout, which exhibited a pressure sensitivity of 1.18 × 10^5^ kHz^3^/kPa that was by far superior to that represented by the cavity length changes for the same F-P sensor. The results validated the developed F-P resonator and the measured resonance frequencies in contrast to *Q* factors revealed the more strongly dependence upon the stress tensor in nanomembranes [27], in comparison to viscous damping or free molecule flow damping previously reported in [28]. 

## 2. Fabrication and Optical Interferometric Readout of the F-P Resonator

**Figure 1:**
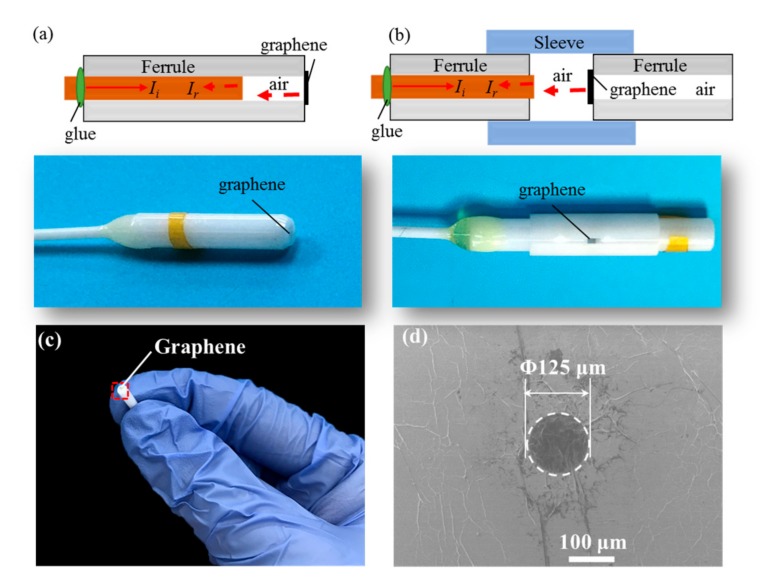
Schematic diagram and physical picture of the F-P sensor samples with (**a**) sealed and (**b**) open cavities (not to scale); (**c**) ~13-layer graphene-covered ZrO_2_ ferrule; and (**d**) the magnification SEM image of the rectangular area shown in (**c**).

Figure 1a,b show the schematic diagram and physical picture of the fabricated F-P resonators with sealed and open cavities. Other than the conventional F-P micro air-cavity in Figure 1a, the sensor sample in Figure 1b introduces another ferrule and a ZrO_2_ mating sleeve onto whose endface the graphene film, working as a light reflector, is adhered by van der Waals forces. The initial cavity lengths between the fiber end and ferrule endface for the two types of samples are confirmed by an optical spectrum analyzer (OSA) (AQ6370C, Yokogawa Electric Corporation, Tokyo, Japan) via a 1-μm resolution translation stage. Then, the magnification scanning electron microscope (SEM, JSM-7500F, JEOL Ltd., Akishima, Japan) image of as-transferred multilayer film on the ceramic ferrule with a 125-μm diameter hole, as shown in Figure 1c, is given in Figure 1d. After that, the single-mode fiber (SMF, Langpu Da Optoelectronics Technology Co., Ltd., Beijing, China) and the ferrule are bonded together by an epoxy adhesive (3M^®^). Note that the procedure of preparing the film from a commercial CVD 10–15-layer graphene sample (ACS Material^®^, Nanjing XFNANO Materials Tech Co., Ltd., Nanjing, China) and transferring it onto the endface of a ferrule is same as that in [29]. The thickness of a piece of graphene sheet transferred on a silicon substrate is measured to be ~4.13 nm by atomic force microscope (AFM, SPA 300HV, Seiko Instruments Inc., Chiba, Japan) with tapping mode, close to roughly 13 layers of graphene with a monolayer thickness of 0.335 nm [30]. 

In [31], Bunch et al. made graphene-sealed square microchambers and measured the pressure-deflection characteristics of graphene membranes using AFM for the first time, which showed the impermeability of transferred membranes was concerned with self-tensioning in graphene resonators due to adhesion to the sidewalls caused by the van der Waals forces [10]. Therefore, the constructed sealed cavity in Figure 1a is employed to balance the pressures at both sides of membrane for evaluation of the influence of air pressure and self-tensioning in air damping. Referring to the multiple-beam interference theory, along with the lower reflectivity (~1.71%) of the ~13-layer graphene [29,32], the reflected intensity *I*_r_ in the F-P cavity in Figure 1a can be approximated as [33]:

(1) $$I_{r}=\left(R_{2}+\xi R_{1}+2\sqrt{\xi R_{1}R_{2}}\cos\theta \right)I_{i},$$

where *R*_1_ and *R*_2_ are the reflectivities of the graphene membrane and fiber endface, respectively; *I*_i_ is the incident intensity; *θ* is the phase difference between two adjacent beams, and *ξ* is the coupling coefficient of cavity length loss [29]. *R*_2_ is confirmed as 2.5% by measuring the interference of F-P cavity formed by two SMFs. As the lattice expands or contracts because of laser power-induced temperature change, the equilibrium positions of atoms and consequently the interatomic forces change, which induces the shift in the phonon energies and then membrane motion [34]. Assuming that the displacement of the diaphragm vibrating axially with simple harmonic motion is $\delta_{\mathrm{mem}}=A\sin\left(2\pi ft+\varphi \right)$, where *t* represents time; *A*, *f*, and *ϕ* are the vibration amplitude, frequency, and phase angle, respectively. According to the resonance motion of diaphragm, *I*_r_ in Equation (1) can be rewritten as:

(2) $$I_{r}=\left[R_{2}+\xi R_{1}+2\sqrt{\xi R_{1}R_{2}}\cos\left(\theta +4\pi \delta_{\mathrm{mem}}/\lambda \right)\right]I_{i},$$

where *λ* is the wavelength of incident light. 

From Equation (2), the resulting motion (*δ*_mem_) of the suspended structure will modulate the reflectivity of the F-P interferometer created by both the graphene membrane and SMF. Since *δ*_mem_ creates a time-dependent variation of tension within the membrane, the *δ*_mem_-related incident light intensity needs to be confined to an appropriate range rather than an undesirable higher light intensity, which otherwise will cause nonlinear mode coupling and internal resonances related with complex energy transfer between various vibrational modes [20,26]. Below a certain threshold energy for obtaining the resonance with a better profile, these modes can be decoupled, thereby leading to comparatively low decay rates and giant quality factors [35]. Actually, the resonance frequency and *Q* factor of the mechanical resonators are closely related with the dimension and physical properties of the resonator material, such as pre-tension, mass density and Young’s modulus, in addition to damping conditions. In this case, the resonance behaviors of pressurized graphene in air can be determined by extracting optically-actuated thermally-induced membrane displacements perpendicular to the graphene diaphragm.

## 3. Experiment and Analysis

**Figure 2:**
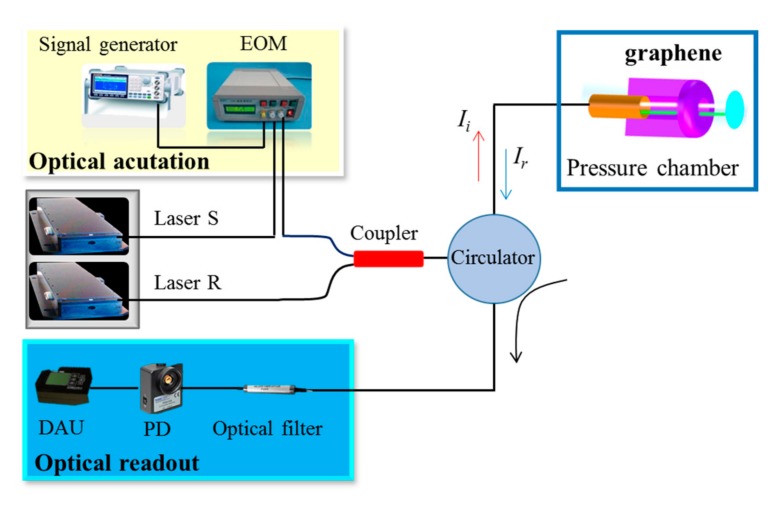
Schematic illustration of optical fiber F-P interferometry setup for resonance measurements.

Figure 2 illustrates the optical fiber F-P interferometric actuation and read-out scheme. A 1550.12-nm amplitude-modulated distributed feedback (DFB) laser S (10 dBm output power, Beijing Conquer Optics Science & Technology Co., Ltd., Beijing, China) was used to impose a point-type thermoelastic excitation upon the suspended graphene diaphragm by modulating the laser intensity through the photothermal expansion and contraction of diaphragm via an electro optic modulator (EOM) (Beijing Conquer Optics Science & Technology Co., Ltd., Beijing, China) with power amplification. The EOM offered the frequency modulation via a signal generator (DG5102) (Beijing, China). Then, another 1551.72-nm DFB laser R (10 dBm output power, Beijing Conquer Optics Science & Technology Co., Ltd., Beijing, China) was employed to pick up the resulting membrane motion. The light signals emitted by the lasers S and R were optically coupled through a 2 × 1 coupler followed by the input into the sensor probe via a three-port circulator so that the power of the laser light delivered to the sample was less than 7.53 μW. The time-variable local heating resulted from the modulated laser S caused a modulated stress due to thermal expansion, thereby generating the driving force exciting the membranes into resonance. Thus the reflected light intensity *I*_r_ would occur to change accordingly, when the resonator vibrated. After separating the reflected light with a wavelength of 1551.72 nm via an optical filter, the light intensity was detected by a 200 MHz bandwidth photodetector with a preamplifier. To evaluate the effects of pre-stress and air damping on resonance characteristics, two types of F-P sensors with open and sealed air-cavity configurations were put inside a pressure chamber. All resonance measurements were performed at room temperature. Firstly, applied pressures were set as 10^5^ Pa (atmospheric pressure) and 10^−2^ Pa (vacuum pressure), where viscous damping was remarkably different since the Knudsen number *K*_n_ was confirmed as around 0.0006 and 6350, respectively. For the former, viscous damping becomes significant. On the contrary, the latter is not in the viscous damping regime. Then regarding the limited impact of air damping on resonance behaviors, the conventional F-P sensor probe was also utilized to further investigate the pressure-sensitive resonance behaviors at pressures ranging from 101 to 300 kPa, i.e., a relative pressure range of 0–199 kPa, with a corresponding lower *K*_n_ value ranging from 0.0002 to 0.0006.

### 3.1. Effect of Membrane Stress on f and Q

**Figure 3:**
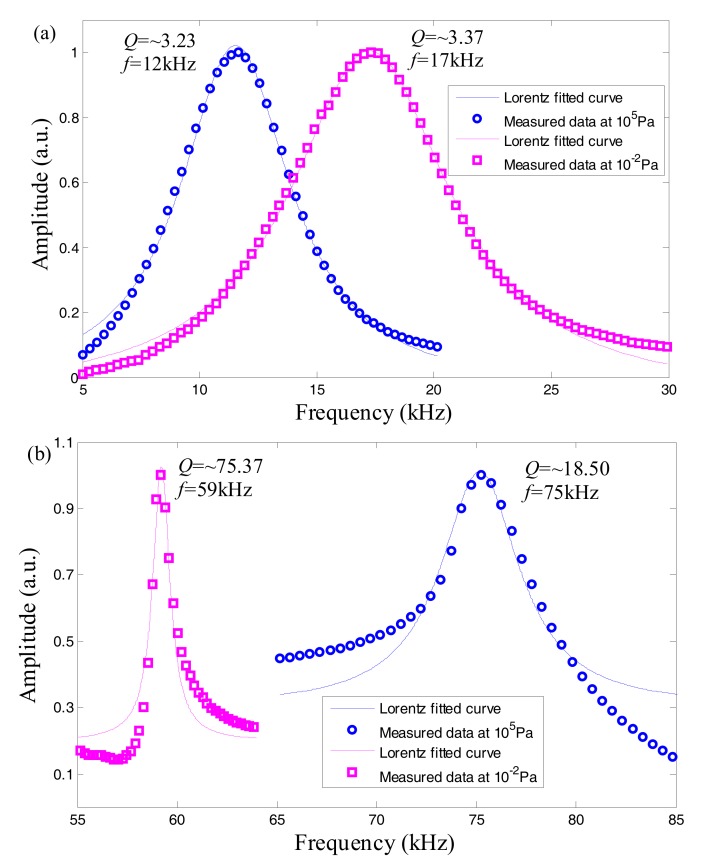
Resonance fundamental frequency *f* and *Q* factor at 10^5^ and 10^−2^ Pa for the sensor probes with (**a**) open and (**b**) sealed cavity structures, respectively.

The photothermal displacement spectrum from the graphene resonator contributes to analyzing the dependence on the primary effect suffering from the membrane thickness-dependent bending rigidity or initial pre-tension. Figure 3 reveals the fundamental frequency *f* and *Q* of the two clamped circular graphene diaphragms at two typical pressures (10^5^ and 10^−2^ Pa) representing viscous and free molecule flow damping regimes because of *K*_n_ = 0.0006 and 6350, respectively. Note that the two diaphragms are correspondingly adhered to the ferrule endfaces in the two types of sealed and open F-P probes as mentioned in Figure 1a,b.

For the F-P resonator with open cavity configuration, no pressure gradient exists within the membrane, which indicates that the mechanical response in Figure 3a is dominated by the surface tension induced by pre-stress rather than the bending tension of pressurized membrane. For this reason, the corresponding fundamental frequency acts as a slight increase in kHz due to the decreasing viscous damping. In previous studies, measured *Q* factor of graphene-based nanomechanical resonators spans from 2 to 2 × 10^5^, as it is strongly dependent on the sample fabrication and measurement conditions [36]. Herein, there is no obvious change in *Q* (around 3.2–3.4) when the air pressure is reduced to 10^−2^ Pa from an atmospheric pressure, thereby implying a lower pre-stress value which is closely concerned with the interfacial energy between graphene and supporting substrates. In accordance with the membrane-to-plate behavior of 2D mechanical resonators with certain thickness [7], the resonance frequency shift of circular graphene film in contact with air in a cross-over regime can be written as [37]:

(3) $$f_{\mathrm{air}}\approx \sqrt{f_{\mathrm{mem}}^{2}+f_{\mathrm{plate}}^{2}}/\sqrt{1+\rho_{\mathrm{air}}r\Gamma /\left(\rho t\right)}=\sqrt{\frac{\frac{0.146}{r^{2}}\frac{S}{\rho t}+\frac{0.22Et^{2}}{\rho \left(1-\upsilon^{2}\right)r^{4}}}{1+\rho_{\mathrm{air}}r\Gamma /\left(\rho t\right)}},$$

where *f*_air_, *f*_mem_ and *f*_plate_ are the resonance frequency in air damping, XXX, and XXX, respectively; *ρ* is the mass density of graphene with certain adsorbates; *ρ*_air_ is the density of air; *r*, *S*, *E*, *t*, and *υ* are the radius, tension, Young’s modulus (1 TPa), thickness and Poisson’s ratio (~0.17) of graphene, respectively; Γ is a non-dimensionalized added virtual mass incremental factor as a function of mode shapes and boundary conditions. Note that Γ = 0.746 in the fundamental resonant mode *f*_0,1_. Hence, the resonance frequencies at various pressures enable the estimation of *ρ* and *S* simultaneously by fitting measured frequencies, along with the established continuum model. In this way, we estimated the pre-tension *S*_0_ to be 1.32 × 10^−5^ N/m for the resonator mentioned in Figure 3a, along with the density *ρ* = 10*ρ*_g_, where *ρ*_g_ is the mass density of pure graphene. The resulting equivalent mass density is similar to graphene resonators in [11,12,13], where the extra mass is attributed to the adsorbates and residues from the transferring and fabrication process.

**Figure 4:**
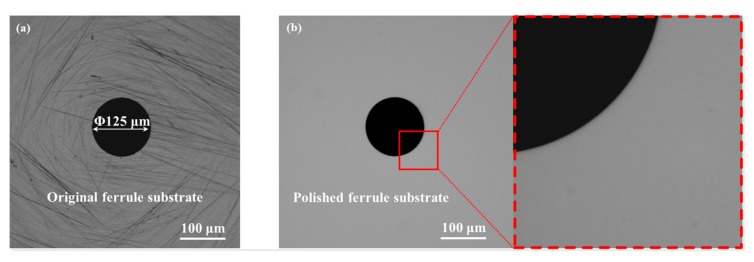
The micrograph of a ferrule endface (**a**) before and (**b**) after polishing (magnification of 100×). Inset: The magnification image of the rectangular area (magnification of 600×).

In contrast, the F-P resonator with sealed cavity configuration in Figure 3b has a higher *Q* factor of ~18.5 with a higher pre-tension *S*_0_ of 0.022 N/m at normal pressure on basis of the aforementioned model. Furthermore, the *Q* factor increases to ~75.37 by lowering the atmospheric pressure to 10^−2^ Pa, although the value is much less than those reported in previous literature [36]. A likely explanation for this phenomenon is that these resonators feature, in μm-order dimension, via a mechanical peeling process without capillary forces involved, and the measurement at vacuum pressures of <10^−5^ Torr [10,11,12,13,17,19] or at low temperatures (<50 mK) [11,16,38]. It is important to point out that the mechanically exfoliating process is beneficial to offering higher pre-tension on account of less intrinsic defects in nanomembranes and favorable substrate adhesion. Nevertheless, graphene prepared from this method is small-size, uneven thickness and difficult to scale the process to mass production. Additionally, the micro-air-gap-based resonator decreases *f* under tensile tension, which conforms to the trend obtained in Equation (3). The small frequency shift of about 15 kHz also partly results from the limited pre-tension. As a result, the benefit of reduced air damping with decreasing pressure will gradually become remarkable, particularly for the resonator with greater pre-tension. Note that the tension is primarily caused by the fabrication process, where the friction between the graphene and the oxide surface stretches the graphene sheets across the trench or hole [10]. Therefore, in order to boost the pre-tension-dependent interfacial behavior, the endface of ZrO_2_ ferrule-supporting suspended graphene was polished with diamond sheets in different grit sizes of 3 and 1 µm (Thorlabs^®^ LF3D, LF1D, Newton, NJ, USA). The residuals on polished surface were cleaned by ultrasound equipment. Figure 4a,b display the micrograph (microscope camera: Mshot^®^, MS60, Guangzhou, China) of a ferrule endface before and after polishing. Small burrs and impurities at the edge of circular hole can be clearly found in Figure 4a, as well as stains and scratches on substrate surface. After surface polishing and cleaning (Figure 4b,c), the ferrule substrate appeared a flat and smooth surface with a surface roughness of less than 1 µm. By this means, the processed ferrule was used as a membrane transfer substrate to optimize the surface tension-dominated resonance responses to applied varying external pressures. However, it should also be added that the polished ultra-flat surface will possibly sharpen the hole edge in the ferrule, thereby causing the partial tiny breakage of graphene membrane adhered on the edge of the hole as well as the non-uniformly stress distributions in membrane. Furthermore, these defects will weaken the clamped boundary conditions of the circular diaphragm, therefore worsening the resonance performance. Therefore, to overcome the problem, a circular edge grinding process around the hole would be introduced in the following study to increase the production yield in high-quality graphene film transferring for experimentally evaluating the variation of the initial tension in membrane with respect to the same type of polishing. Additionally, it can be concluded that the initial tension will occur to vary within a certain range, in view of the undesirable factors involving uncontrollable manual grinding and non-uniform graphene sheets with the adsorbates and residues from the transferring and fabrication process.

### 3.2. Pressure-Sensitive Resonance Responses in Air Damping

**Figure 5:**
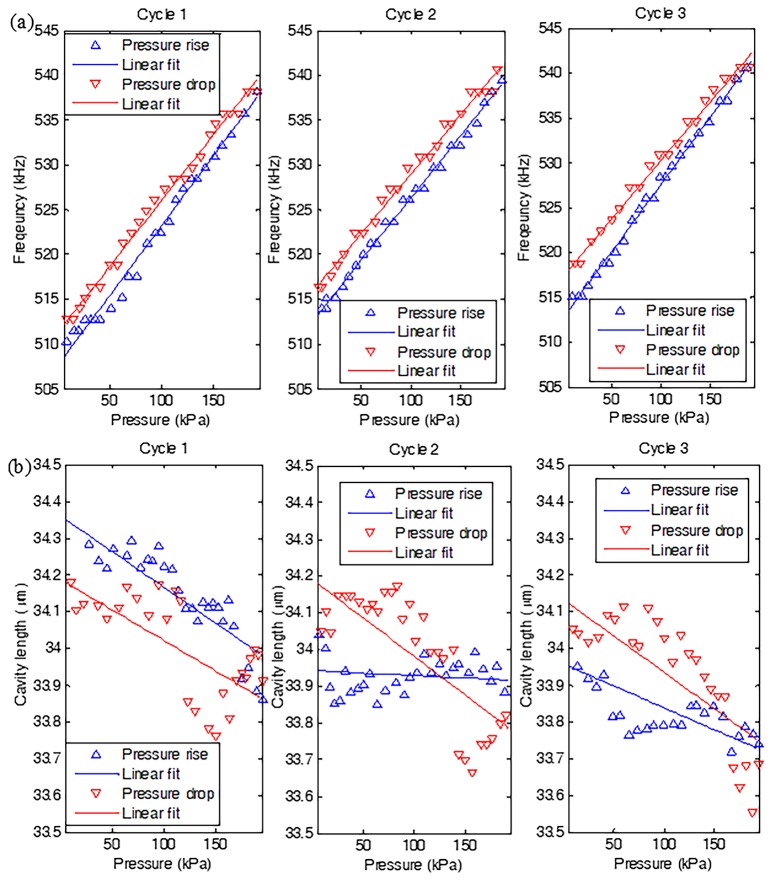
Applied pressure verse (**a**) resonance frequency and (**b**) cavity length for an F-P sensor with a sealed micro air-cavity at three cycles of pressure rise/drop measurements.

Referring to Figure 2 again, the sealed F-P resonator with multilayer graphene suspended onto the polished ferrule is placed in the pressure chamber. Then the resonance frequency versus external pressure was imposed on the graphene diaphragm at three cycles of pressure rise/drop measurements in the relative pressure range of 0–199 kPa is given in Figure 5a, where the three measurements in the positive direction are in good agreement with those in the negative direction. However, there exists hysteresis errors of 5.98%, 6.03%, and 7.69%, respectively, which is, to a great extent, caused by the non-uniformly distributed adsorbates [11] and the air pressure fluctuation in the sealed F-P cavity covered with imperfect graphene membrane occurring during a wet CVD graphene membrane transfer, such as microscopic wrinkles and surface defects [39,40]. As a consequence, the pressure reverted to an inconsistent value at different cycles of pressure drop measurements. Based on the measured average frequencies during the whole measurements, the pressure sensitivity of the F-P sensor was fitted to be ~142.2 Hz/kPa by using a least square fitting method with a good fitting *R*-square of about 92.7%. Additionally, in terms of small fluctuations relative to the fitted output, a decent linearity of 5.15% was obtained due to fast dynamic response of the suspended membrane. After that, a conventional F-P interferometric readout scheme was also performed for the fabricated sensor to evaluate the presented F-P resonant detection. Referring to Figure 5b, the decreased cavity length was roughly compatible with each other at three cycles of pressure drop measurements, despite the presence of more obviously-scattered data. Thus, a fitted low average pressure sensitivity of 1.91 nm/kPa was achieved with a poor fitting *R*-square of 53.5% in the negative going direction. Unfortunately, the sensor demonstrated a more inferior response in the positive going direction. It can be clearly observed that the measured three time results deviated from each other, particularly at the second cycle where an almost zero response occurred. For the remaining two cycles of positive measurements, an extremely low sensitivity of around 1.57 nm/kPa was recorded. It should be pointed out that during the F-P interferometric experiment, a broadband laser source (ALS-CL-17, Amonics Ltd., Hong Kong, China) was used to illustrate the sensor, and the reflection spectrum was then monitored by an OSA (AQ6370C) with a wavelength resolution of 0.02 nm. With regard to the clamped circular graphene film made of a linear isotropic elastic material, based on the spherical shell equation, the relationship between the diaphragm deflection *ω* and the pressure change *P* is defined by [32]:

(4) $$P=\frac{4\sigma_{0}t}{r^{2}}\omega +\frac{8Et\omega^{3}}{3\left(1-\upsilon \right)r^{4}},$$

where *σ*_0_ is the pre-stress of graphene that was confirmed as ~0.24 GPa on basis of the measured resonance frequency. Then a theoretical pressure sensitivity of 1121 nm/kPa was solved according to Equation (4), which exceeded, by far, the aforementioned measured 1.91 or 1.57 nm/kPa. The resulting load-deflection behavior primarily resulted from a lower pressure difference that was concerned with the slow air leakage of F-P cavity covered with large-sized CVD multilayer graphene film. Moreover, the phenomenon became more serious in pressure rise measurement than in pressure drop measurement because of longer air balance process in the air chamber. Although recent advances in graphene synthesis and processing have enabled demonstration of atomically-thin 2D membranes showing mechanical sturdiness and hermetic sealing [29,41], a limited size of porous substrate with a pore of 12.56–22.56 μm^2^ in cross-sectional area is generally chosen to reduce the possible risk of leakage from random defects in large-area nanomembranes [42] or poorly-defined peripheral stress distributions on the edge of ferrule hole.

**Figure 6:**
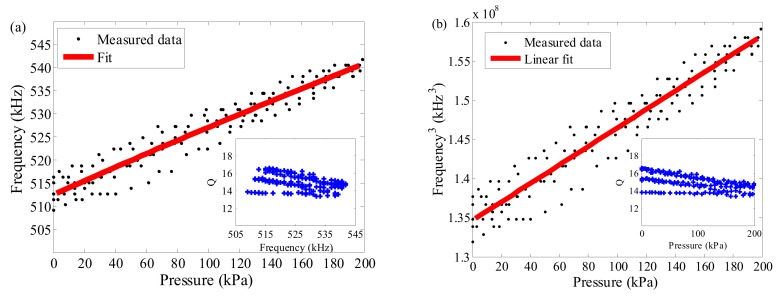
(**a**) Resonance frequency *f* verse pressure. Inset: *Q* factor verse *f*; and (**b**) *f*^3^ verse pressure. Inset: *Q* factor verse pressure.

Figure 6 reveals the variation of measured resonance characteristics (*f* and *Q*) over the tested pressure range. Owing to the rising pre-stress tuned by the polished substrate, the measured *f* was increased to more than 500 kHz well above the frequency of 60–80 kHz in Figure 3, thus showing the fundamental frequency response ranging from 509 to 542 kHz as shown in Figure 6a. In combination with the density assumption of *ρ* = 10*ρ*_g_ mentioned above, an improved pre-tension *S*_0_ for the F-P resonator using the polished surface of a ferrule as film substrate was calculated to be 1.05 N/m. However, a slight frequency deviation up to about 33 kHz existed between them, which is to a great extent due to the presence of non-uniformly distributed adsorbates or polymethylmethacrylate (PMMA) residuals and the stress distributions in the graphene-substrate interface during membrane transfer and sensor fabrication as mentioned above. In the light of the aforementioned Equation (3) relating to *f*_mem_, *f*_plate_ and *f* in the membrane, plate, and cross-over regime, respectively, it can be inferred from Figure 6a that the resonator made of ~13-layer graphene behaved as a circular membrane dominated by the initial pre-tension and pressure difference across the membrane, instead of a commonly assumed plate-like one with negligible initial pre-tension for flakes thicker than 10 layers [7]. Then referring to Figure 6b, a fitted pressure sensitivity of 1.18 × 10^5^ kHz^3^/kPa with a good linearity error of 5.16% was achieved in the tested range, which is far below the theoretical predicted sensitivity of 1.32 × 10^7^ kHz^3^/kPa in terms of the Equation (5) as follows:

(5) $$f_{\left(0,1\right)}{}^{3}\approx \frac{2.404^{3}}{64\pi^{3}}\Delta p\sqrt{\frac{8E}{3\left(1-\upsilon \right)t^{2}\rho^{3}r^{4}}},$$

where *E* and *ρ* are assumed as 1 TPa [43] and 10*ρ*_g_ as mentioned above, respectively. The great deviation between the measured and predicted resonant frequencies possibly results from the equivalent density-dependent impurity and defect effects in transferred suspended graphene with a large membrane dimension, the pre-tensioned-membrane size effect and boundary instability of clamped circular graphene diaphragm in air damping. It should be added that the discrepancy between the measured values and predicted ones is also dependent on the presence of higher eigenmodes featuring the nonlinear oscillation mode coupling with rolling frequency multiplication components, which results from the alternating tension in graphene because of higher vibration amplitude comparable to the thin membrane thickness [33,37,44]. Accordingly, further research on micro-engineering interfacial adhesion interactions using micro-electromechanical systems (MEMS) technology should be focused on strengthening the boundary conditions of the clamped graphene circular membrane [45], thereby enhancing the pressure sensitivity.

Then, according to the inset in Figure 6a, the *Q* factor can be determined by the full width half-maximum of the Lorentzian resonance peak; however, there is no exact dependence of *Q* on resonance frequency. However, as illustrated in the inset in Figure 6b, the *Q* factor decreases as a function of external pressure that is represented by *p*_ext_, approximately proportional to 1/*p*_ext_, which is well in agreement with the variation in [22]. Furthermore, the extracted *Q* factor from ~13.3 to ~16.6 fluctuates around 14.9, which is weakly associated with fundamental frequencies. The average *Q* factor is ~14.9 for the resonator surrounding the air at normal temperature, which is obviously better than that with extremely low pre-tension (1.32 × 10^−5^ N/m) in Figure 3a but roughly close to that with a relative high pre-tension (0.022 N/m) in air damping in Figure 3b. Furthermore, the measured lower *Q* factors are also superior to the results at atmospheric pressure in [21,22]. Then compared with top-down fabricated MEMS resonators, such as silicon nitride nanostrings, which demonstrated a fitted linear relationship between *Q* factors from 17 to 600 and resonant frequencies from 7 MHz to 206 MHz at room temperature [27], the change of *Q* factor for the resonator presented in this paper is relatively significant in the limited test frequency range. Note that it is known that the increase in membrane size enables to reduce *f* and *Q* in viscous damping, but to enhance *Q* in free molecule flow damping. Consequently, engineering structural size-dependent tensile stress in membrane-type resonators can further elevate *Q* by raising instinct energy stored in graphene membranes [44].

It is worth pointing out that the experimental results elucidate that a simple and miniature open or sealed optical fiber F-P pressure-sensitive resonator with large-scale graphene or other 2D diaphragms could be successfully developed without need of complicated free-space optical actuation experimental setups. In order to further elaborate the uniqueness of the developed F-P resonator, some typical experimental works on graphene nanomechancial resonators were summarized and listed in Table 1. It can be clearly observed from this table that the measured resonance frequency and *Q* factor values are by far less than most of the results reported in previous literatures. The resulting primary reason is that those resonators in [10,11,12,15] operate at vacuum pressure where viscous damping is not dominated because *K*_n_ > 10^4^. However, according to the remarkably reduced *Q* factors in reported experimental results, the resonators in [21,22,31] should be in a transition regime between viscous damping and free molecule flow damping, when *K*_n_ decreases to the range of 10–50. Then when *K*_n_ < 0.01, air damping becomes a significant viscous dissipation mechanism, which causes the *Q* factor to drop sharply. Thus, the presented resonator in this paper exhibited a same order of *Q* factor as those working at normal pressure in [21,22,31] and even much better resonance response than the one in [21]. The thin circular membrane dimension (layer number, diameter)-dependent resonance characteristics are dominated by residual tensile stress that can be controlled by graphene-substrate interfacial adhesion behaviors during film transfer and patterned microstructure fabrication. However, it is possible for a resonator with low pre-tension operating in free molecule flow damping to reveal a modest *Q* factor. This suggests that the adhesion conditions at the graphene-substrate interface should be controlled reasonably. Unfortunately, since the adsorbates would generate additional masses and tensions that will result in the dispersion of resonant responses among different samples, the damping mechanism-dependent *K*_n_ should be designed optimally by engineering membranes suspended on F-P resonators. In consideration of the film integration in resonators, the facile-fabricated and in situ F-P resonator is vital for dynamical performance evaluation in actual applications, along with its corresponding simple F-P photothermal actuation and detection scheme. Compared with previous graphene nanomechanical resonators reported in Table 1, the developed resonator inherits the advantages of optical fiber sensors over electrical sensors, and uses a clamped circular diaphragm as a sensitive component rather than a doubly-clamped beam to obtain more predictable resonance frequency. In particularly, regarding the air sealed in conventional F-P sensors, the effects of pressure difference across the pre-stress-dependent graphene diaphragm on resonance responses in air damping are more focused on in this paper. Further research on the strain-tunable substrate [46] for graphene-based F-P resonators is needed to control in situ the in-plane stress for optimizing resonance responses.

## 4. Conclusions 

Two types of simple, miniature, and in situ mechanical F-P resonators with open and sealed cavities were fabricated to characterize the resonance behaviors of a nanothick graphene diaphragm suspended onto a large-area 125-μm inner diameter ferrule endface by using simple optical interference excitation and detection. The effects of gas pressure-induced damping and pre-tension in membranes on resonance characteristics were experimentally investigated in the absolute pressure range of 101 to 300 kPa at room temperature. Due to a smaller pre-tension in membrane, the micro air-cavity-based graphene device exhibited the lower resonance frequency of 59 kHz but remarkable Q factor of ~75.37 at 10^−2^ Pa due to the free molecule flow damping. Then according to the interaction with the air, the pressure-induced modal frequencies from 509 to 542 kHz were further identified with little dependence on Q ranging 13.3–16.6. Despite lower measured Q factors in comparison to the value up to 2 × 10^5^ reported in previous studies where vacuum pressure of <10^−6^ Torr or low temperature of <50 mK were generally set, the experimental results herein demonstrated a pressure sensitivity of 1.18 × 10^5^ kHz^3^/kPa with a linearity error of 5.16%. Although its sensitivity was far below the predicted one possibly because of impurity and defect effects in graphene, pre-tensioned-membrane size effect and boundary instability of clamped diaphragm in air damping, the fabricated graphene-based F-P resonator demonstrated a preferable response to applied static pressures, compared with a conventional F-P interferometer with same micro air-cavity configuration. Therefore, this study could shed light on the resonance behavior in situ observed in 2D nanomechanical resonators in air damping, along with strain-tunable substrate microstructure and thin film vacuum encapsulation.

## Figures and Tables

**Table 1:** Summary of the experimental works on graphene nanomechanical resonators.

Fabrication Method	Layers	Geometry	*f* (MHz)	*Q* Factor	Driving	Readout	Experimental Conditions	*K*_n_	Refs.
Exfoliated	1–143	Doubly-clamped beam	10–170	20–850	Electrical/ Free-space optical	Free-space optical	Room temperature (RT); <1.3 × 10^−4^ Pa	2.8 × 10^7^	[10]
Exfoliated	1	Doubly-clamped beam	30–120	125	Electrical	Electrical	RT; <1.3 × 10^−3^ Pa	2 × 10^6^	[11]
			30–120	14,000			5K; <1.3 × 10^−3^ Pa	3 × 10^4^	
CVD	1	Doubly-clamped beam	5–75	250 (RT), 9000 (10K)	Electrical/ Free-space optical	Electrical/ Free-space optical	RT&10K; <6.7 × 10^−3^ Pa	1 × 10^4^– 3.5 × 10^5^	[12]
Exfoliated	2–5	Doubly-clamped beam	8–23	300–1100	Thermal noise	Electrical	RT; <10^−3^ Pa	7 × 10^5^	[15]
CVD	30–60	Doubly-clamped beam	0.088–0.135	2–81	F-P optical	F-P optical	RT; 10^−2^–10^5^ Pa	6 × 10^−4^– 6 × 10^3^	[21]
Exfoliated	~30	Circular drum	13–17	3–80	Free-space optical	Free-space optical	RT; 10^2^–10^5^ Pa	0.015–15	[22]
Exfoliated	1	Square drum	30–90	25	Free-space optical	Free-space optical	RT; 27–3 × 10^4^ Pa	0.05–54.87	[31]
Exfoliated	<5	Doubly-clamped beam	108–122	/	Electrical	Electrical	RT; <6.7 Pa	525	[46]
CVD	~13	Circular drum	0.509–0.542	13.3–16.6	F-P optical	F-P optical	RT; 10^5^–2.99 × 10^5^ Pa	2 × 10^−4^– 6 × 10^−4^	This work
